# Hemoglobin A_2_ and Heterogeneous Diagnostic Relevance Observed in Eight New Variants of the Delta Globin Gene

**DOI:** 10.3390/genes12111821

**Published:** 2021-11-19

**Authors:** Noraesah Mahmud, Massimo Maffei, Massimo Mogni, Gian Luca Forni, Valeria Maria Pinto, Giuseppina Barberio, Silvana Ungari, Antonella Maffè, Cristina Curcio, Francesco Zanolli, Raffaella Paventa, Mariarosa Carta, Alberta Caleffi, Mariella Mercadanti, Sauro Maoggi, Giovanni Ivaldi, Domenico Coviello

**Affiliations:** 1 Laboratorio Genetica Umana, IRCCS Istituto Giannina Gaslini, 16147 Genova, Italy; noraesah.mahmud@moh.gov.my (N.M.); massimomogni@gaslini.org (M.M.); domenicocoviello@gaslini.org (D.C.); 2 Department of Pathology, Hospital Kuala Lumpur, Kuala Lumpur 50586, Malaysia; 3 Centro Microcitemia e Anemie Congenite, Ospedali Galliera, 16128 Genova, Italy; gianluca.forni@galliera.it (G.L.F.); valeria.pinto@galliera.it (V.M.P.); 4 Medicina di Laboratorio, Azienda ULSS 2 Marca Trevigiana, 31100 Treviso, Italy; giuseppina.barberio@aulss2.veneto.it; 5 Genetica e Biologia Molecolare ASO S. Croce e Carle, 12100 Cuneo, Italy; ungari.s@ospedale.cuneo.it (S.U.); maffe.a@ospedale.cuneo.it (A.M.); 6 Fondazione IRCCS Ca’ Granda Ospedale Maggiore Policlinico, 20122 Milano, Italy; cristina.curcio@policlinico.mi.it; 7 Formerly, Servizio di Immunoematologia e Medicina Trasfusionale, A.O. Santa Maria degli Angeli, 33170 Pordenone, Italy; francesco.zanolli@hotmail.it; 8 Formerly Laboratorio Analisi, ASL AT, 14100 Asti, Italy; raffaellapaventa@gmail.com; 9 Medicina di Laboratorio, Ospedale S. Bortolo, AULSS 8 Berica, 36100 Vicenza, Italy; mariarosa.carta@aulss8.veneto.it; 10 U.O. Diagnostica Ematochimica, Azienda Ospedaliero-Universitaria, 43126 Parma, Italy; acaleffi@ao.pr.it (A.C.); mercandrea1953@libero.it (M.M.); 11 Sebia-Italia S.r.l., 50012 Bagno a Ripoli, Italy; sauro.maoggi@sebia.it; 12 Formerly, Laboratorio Genetica Umana, Galliera Hospital, 16128 Genova, Italy; g.ivaldi@live.it

**Keywords:** δ-globin gene, δ-globin gene variant, Hb A_2_-variant, β-thalassemia, Hb-variants

## Abstract

Background: Hemoglobin A (Hb A) (α_2_β_2_) in the normal adult subject constitutes 96–98% of hemoglobin, and Hb F is normally less than 1%, while for hemoglobin A_2_ (Hb A_2_) (α_2_δ_2_), the normal reference values are between 2.0 and 3.3%. It is important to evaluate the presence of possible delta gene mutations in a population at high risk for globin gene defects in order to correctly diagnose the β-thalassemia carrier. Methods: The most used methods for the quantification of Hb A_2_ are based on automated high performance liquid chromatography (HPLC) or capillary electrophoresis (CE). In particular Hb analyses were performed by HPLC on three dedicated devices. DNA analyses were performed according to local standard protocols. Results: Here, we described eight new δ-globin gene variants discovered and characterized in some laboratories in Northern Italy in recent years. These new variants were added to the many already known Hb A_2_ variants that were found with an estimated frequency of about 1–2% during the screening tests in our laboratories. Conclusions: The knowledge recognition of the delta variant on Hb analysis and accurate molecular characterization is crucial to provide an accurate definitive thalassemia diagnosis, particularly in young subjects who would like to ask for a prenatal diagnosis or preimplantation genetic diagnosis.

## 1. Introduction

Adult hemoglobin (Hb A) (α_2_β_2_) accounts for 96–98% of all functioning hemoglobin in adult life. Fetal hemoglobin (Hb F) (α_2_γ_2_) is prevalent in fetal life, but decreases after birth to a normal of less than 1% by the first year of life [1]. At the same time, there is synthesis of hemoglobin A_2_ (Hb A_2_) (α_2_δ_2_), which after one year of age represents a minor component of circulating hemoglobin, and in the normal adult individual is present in an amount of less than 3.2%. It has been agreed that the reference range for Hb A_2_ in normal subjects generally ranges from 2.0 to 3.3%, but it has also been observed that it may differ slightly, depending on the method of quantification and in different population groups [2,3]. Considering the small percentage differences that may exist between normal and pathological Hb A_2_ values, high precision in the measurement of this important parameter is required. There are several methods for testing Hb A_2_, but the two most widely used are automated high-performance liquid chromatography (HPLC) and capillary electrophoresis (CE) [4,5]. The differences between the available commercial separation methods have still proven to be significant, even though the use of switchable control materials can halve the variability between methods [6]. For this reason, the International Federation of Clinical Chemistry (IFCC) has launched a standardization program with the goal of establishing a comprehensive reference system for Hb A_2_ measurements [7,8]. However, it is recommended that each laboratory establish its own normal Hb A_2_ ranges based on their population and methodology employed. The Hb A_2_ level is certainly an important parameter for the presumptive diagnosis of β-thal carrier but this parameter should always be interpreted in accordance with red blood cell indices, morphology, and assessment of the presence of any abnormal Hb components for a more accurate diagnosis. It should be noted that the detection of a β-thal carrier is more complex when the same subject is also a thalassemia carrier or has structural defects of α and δ genes. These types of defects usually lead to a decrease in normal Hb A_2_ levels. Other causes such as severe iron deficiency anemia and sideroblastic anemia could also interfere with the measurement of Hb A_2,_ and these causes should be excluded when interpreting the quantification of the Hb A_2_ result [9]. Defects in δ-globin genes directly affect Hb A_2_ production qualitatively or quantitatively. Most δ variants expressing Hb A_2_-X and δ-thalassemia (δ-thal) are characterized by normal red blood cell indices and are associated with significantly lower levels of unmutated Hb A_2_-Normal. Hb A_2_-X has a peak that can be observed at a different migration than the Hb A_2_-Normal peak. This migration depends on the total charge involved in the amino acid substitution that produces the mutated delta chains. Hb A_2_-X can be “faster” or “slower” than Hb A_2_-Normal, but this finding may also depend on the separation methods used [4]. In some cases, Hb A_2_-X could migrate under HbA, HbF or other possible Hb variants present, or may not be expressed due to structural instability, as could be observed in δ-thal. The δ-variant, when visible and quantified, must be calculated with Hb A_2_-Normal, in order to conclude a diagnosis of β-thal carrier. A structural defect in the δ-chains can express similar or even much reduced Hb A_2_-X compared to Hb A_2_-Normal. A δ-thal mutation would hinder the observation of Hb A_2_-X peak, but it will alter the value of Hb A_2_. This HbA_2_ level will be reduced in relation to the presence of δ^+^ or δ^0^−thal defects and, of course, will also happen in the presence of a β-thal defect [10,11,12,13]. These findings may, therefore, interfere with the accurate diagnosis of β-thal carriers. Therefore, carriers of a δ-variant who are also β-thal carriers may have borderline, normal, or low Hb A_2_ levels. Subjects suspected of having a δ-globin mutation should, therefore, prompt for further molecular testing for a definitive diagnosis, especially when such subjects are examined at a reproductive age or in a preconception setting. The knowledge of mutation identification is important for genetic counselling and prenatal diagnosis or preimplantation genetic diagnosis. An accurate and definitive diagnosis is particularly important in a high-risk population for β-globin, such as in Italy, or when the heterogeneity of β-globin defects may further contribute to a high proportion of individuals with borderline Hb A_2_ [3,14,15,16,17]. Defects in δ-globin genes are expressed in very heterogeneous forms, which are not easily detected by 1st level tests. This contributes to the non-diagnosis of qualitative-quantitative defects of Hb A_2_ and to erroneously consider these defects to be irrelevant in genetic transmission. In fact, a superficial evaluation of the hemoglobin separative test can produce wrong diagnoses of β-thalassemia, as well as misdirect the eventual genetic counselling. Moreover, the absence of specific comments in the first level reports can lead to the frequent decision not to proceed to a molecular confirmation. In the world population, the prevalence of these defects is not well known, but based on our experience, we can estimate that in the Italian population, it is approximately 1–2%. Here, we have described eight new variants of the δ-globin gene, with different phenotypic features, observed and characterized in Northern Italy during the last few years. These cases contribute to the knowledge of new features in addition to the many existing δ-globin gene defects reported in dedicated databases.

## 2. Materials and Methods

The samples of the 12 subjects described in this study were sent by different centers in Parma, Genoa, Asti, Pordenone, Milan, Treviso, and Cuneo. All patients provided their informed consent. Complete blood count, iron status, Hb analysis and DNA analysis were performed according to local standard protocols by the respective laboratories. In particular, Hb analyses were performed by HPLC on three dedicated devices: VARIANT II^TM^ β-Thalassemia short program, VARIANT II^TM^ Dual Kit program, and D-10 (Bio-Rad Laboratories, Hercules, CA, USA), and also by CE (Capillarys 2 Flex Piercing; Sebia, Lisses, France). Genomic DNA was isolated using the Qiagen Mini Blood DNA kit (Qiagen GmbH, Hilden, Germany). α-thalassemia (α-thal) analyses were performed using α-Globin StripAssay ™ (ViennaLab Diagnostics GmbH, Vienna, Austria) [18].

The sequencing of the δ-, β-, and α-globin genes was performed with the Sanger method, using dedicated primers [19]. Specific primers for delta gene sequencing are described by Olds RJ et al. [20].

The δ variants have been named as reported in HbVar [21] following the classic nomenclature and as indicated by HGVS recommendations [22].

## 3. Results

**Figure 1:**
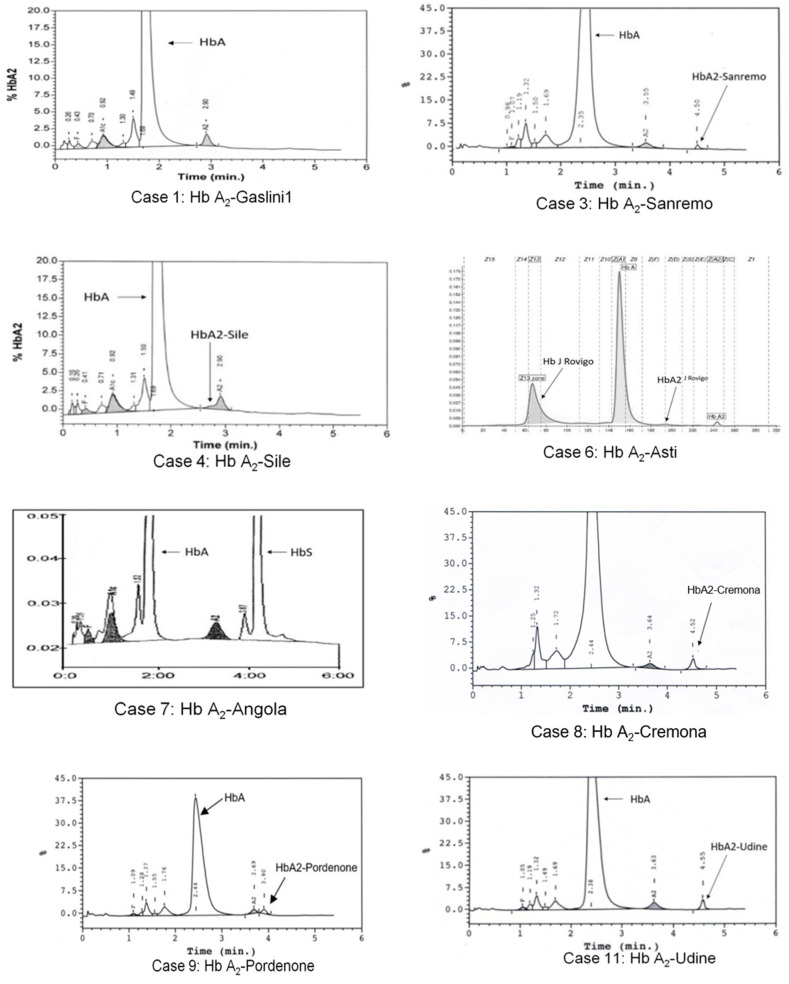
HPLC and CE profiles present in the eight subjects with Hb A_2_ variants: case numbering corresponds to that shown in Table 1. Case 6 shows the profile in which the Hb A_2_-Asti or (α_2_δ_2_^Asti^) is not visible as co-migrating with Hb A. Instead, we can observe Hb A_2_^J-Rovigo^ or (α_2_^J-Rovigo^ δ_2_) is not quantifiable, but visible as a basic movement in the “Hb F zone” and produced by the presence of α^J-Rovigo^ chains that, with the normal delta chains, form a mutated tetramer. No Hb A_2_ variant can be observed for cases 1 and 7.

**Figure 2:**
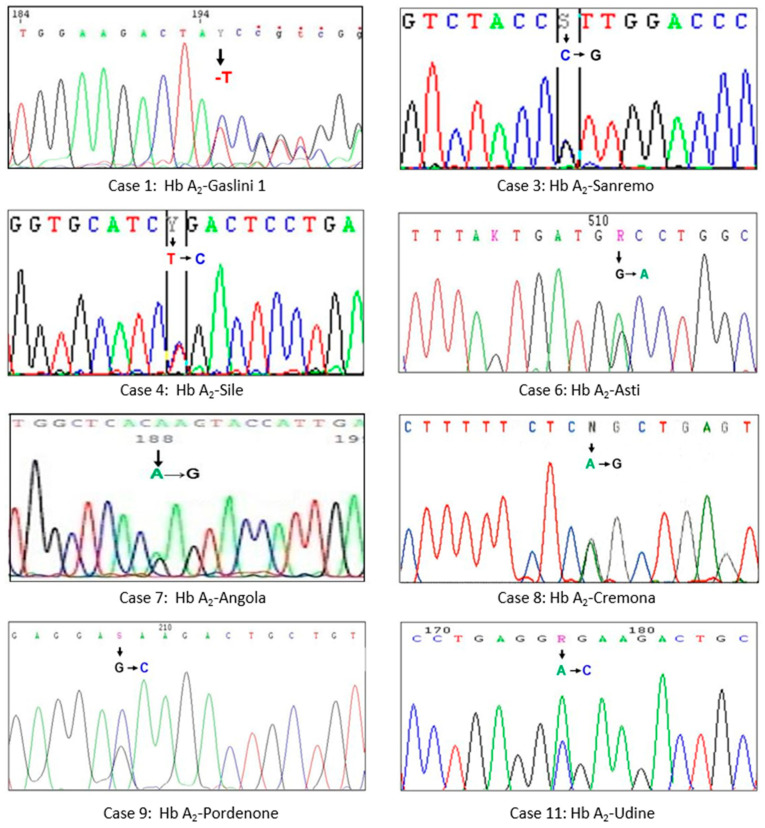
Parts of the DNA sequencing of the δ gene showing the substitutions of a single nucleotide (arrow) in the eight Hb A_2_ characterized variants: the numbering of cases corresponds to that reported in Table 1.

The hematological parameters and molecular results of the 12 examined subjects belonging to eight different families are shown in Table 1; the relative value of Hb F was within the normal range (<1%) in all examined subjects. Chromatographic or electrophoretic profiles are shown in Figure 1, and electropherograms of nucleotide sequences are in Figure 2. In the following, for each characterized variant, we illustrate the results of the study performed with commentary useful for their interpretation, referring to the data in Table 1. Every single mutation detected was correlated with the erythrocyte indices and the respective chromatographic or electrophoretic behavior, considering the pH conditions of the systems used, as well as the electrical charges of the amino acids involved. In addition, the homology between the substitutions documented in the delta chains, and those corresponding to and more often expressed in the α and β chains reported in the literature, were particularly considered.

### 3.1. Hb A_2_-Gaslini 1

Case 1. A 35-year-old Brazilian woman, during tests for the prevention of hemoglobinopathies, showed a reduced value of Hb A_2_ (1.6%), and no alteration of the red blood cell indices. Sequencing of the δ globin gene revealed the deletion of a nucleotide base (-T): Hb A_2_-Gaslini1 δ130 (-T) (*HBD*:c.391delT; p.Tyr131Ilefs*?). The defect can be classified as a thalassemic variant δ or new δ0-thal. The deletion of the nucleotide T in the first position of codon 130 (TAT > ↓ATC), that is, in exon 3, 17 aminoacidic residues before the wild type stop codon, leads to a consequent change of Tyr to Ile, loss of the original stop codon (TGA), lengthening of the δ-globin chain with additional 66 acid amino (aa) residues, and generation of the new stop codon (TAG). These residues consist of four cysteine that lead to protein instability. In accordance with HGVS recommendations [22], the name for this variant is indicated as fs*? because the new reading frame does not meet a new stop codon in the immediate vicinity. Only by doing a manual analysis of the sequence we were able to identify the new termination codon. This is the second defect of the δ gene produced by the deletion of a single nucleotide [20]; other similar cases are known, but concern the β gene.

### 3.2. Hb A_2_-Sanremo

Cases 2 and 3. The proband (Case 2), a 26-year-old woman presenting with an iron deficiency anemia, had a reduced level of Hb A_2_ (1.5%), and no other abnormal peaks. The father (Case 3) had the same Hb A_2_ value as the daughter, but also exhibited presence of a minor peak (0.5%), presenting no anemia. The analysis of the δ-globin gene sequence of both subjects highlighted the presence of the transversion C > G in exon 2: Hb A_2_-Sanremo δ36 (C2) Pro → Arg (*HBD*:c.110C > G; p.Pro37Arg). This variant in the proband was not quantifiable due to iron deficiency anemia.

### 3.3. Hb A_2_-Sile

Case 4. A δ variant was suspected in an asymptomatic 27-year-old Albanian woman with a normal blood count in pregnancy. HPLC revealed a reduced (1.7%) and asymmetric Hb A_2_ peak. Molecular analysis of the δ-globin gene showed a T > C transition in exon 1: Hb A_2_-Sile δ3 (NA3) Leu → Pro (*HBD*:c.11T > C; p.Leu4Pro). This variant is probably unstable, but this property is difficult to document with the classic tests, as were the cases in α and β variants, with replacement Leu → Pro, present in the literature [23,24].

### 3.4. Hb A_2_-Asti

Case 5. A 10-year-old Italian boy showed altered erythrocyte indices and normal Hb A_2_ during screening tests. Molecular analysis excluded the presence of α-thal, but direct sequencing of the β-globin gene revealed a β-thal heterozygosity: (β0 cod39(C > T); (*HBB*:c.118C > T)). Since a Hb A_2_ value not compatible with β-thal was present, sequencing of the δ-globin gene was performed which revealed a new mutation called Hb A_2_-Asti δ74 (E18) Gly → Asp (*HBD*:c.224G > A; p.Gly75Asp). This result allowed us to attribute to the variant Hb A_2_ an electric charge similar to Hb A and, therefore, not detectable.

Case 6. A clinically asymptomatic 45-year-old Italian man examined by CE revealed a more electronegative variant (26.2%) of Hb A and a reduced amount of Hb A_2_ (1.0%). Analysis of the α-globin gene showed the presence of a heterozygous variant: α53 (E2) Ala → Asp; (*HBA2*:c.161C > A) (Hb J-Rovigo), while the sequenced δ-globin gene showed the Hb A_2_-Asti variant δ74 (E18) Gly → Asp (*HBD*:c.224G > A; p.Gly75Asp). Normal Hb A_2_ was reduced compared to Case 5, due to the following fragmentation: Hb A_2_-Normal or (α_2_δ_2_) (1%) and Hb A_2_-Asti or (α_2_δ_2_^Asti^) were not visible as co-migrants with Hb A, Hb A_2_^-J Rovigo^ or (α_2_^J Rovigo^ δ_2_) was not quantifiable, but visible as a basic movement in the “Hb F zone” (Figure 1), and a hybrid tetramer (α_2_^-J Rovigo^ δ_2_^-Asti^) was not visible because it is produced in a quantity lower than the sensitivity threshold of the instruments used.

### 3.5. Hb A_2_-Angola

Case 7. A 33-year-old Angolan woman with a normal blood count, already known as a Hb S carrier, had reduced Hb A_2_. Sequencing of the δ-globin gene showed a new heterozygous variant: Hb A_2_-Angola δ144 (HC1) Lys → Glu (*HBD*:c.433A > G; p.Lys145Glu). The replacement Lys → Glu involves the elution of Hb A_2_-Angola with Hb A.

### 3.6. Hb A_2_-Cremona

Case 8. An abnormal peak (1%) and a decrease in Hb A_2_ (1.5%) in HPLC were observed during routine Hb analysis in a 40-year-old woman of Lombard origin. Analysis of the δ gene sequence revealed an A > G transition in exon 2: Hb A_2_-Cremona δ87 (A4) Gln → Arg (*HBD*:c.263A > G; p.Gln88Arg).

### 3.7. Hb A_2_-Pordenone

Case 9. A variant δ was observed in a 62-year-old woman of Friulan origin with mild microcytosis not due to iron deficiency and in which the presence of α-thal was excluded. Analysis of the δ-gene sequence revealed a new G > C transversion in exon 1: Hb A_2_-Pordenone δ7 (A4) Glu → Asp (*HBD*:c.24G > C; p.Glu8Asp).

### 3.8. Hb A_2_-Udine

Cases 10, 11 and 12. Three family members belonging to three different generations (propositus (Case 11), his mother (Case 10), and his daughter (Case 12)), natives of the Friuli region, were examined to ascertain the possible presence of β-thal. The propositus presented with hypochromic microcytic without iron deficiency. In HPLC, all three subjects had normal Hb A_2,_ or less than 2%, and had an additional peak after Hb A_2_. The additional peak values were significantly higher in the propositus than in his family members. The α, β and δ genes of all three subjects were examined and a heterozygous β-thal was observed in the propositus (Case 11) ((β0 cd39(C > T); *HBB*:c.118C > T)), associated with Hb A_2_-Udine δ7 (A4) Glu → Ala (*HBD*:c.23A > C; p.Glu8Ala). The other two family members (Cases 10 and 12) were carriers of only the new δ variant.

## 4. Discussion and Conclusions

This report described eight new hemoglobin delta variants that have not been published previously, that highlight the importance of identifying delta variants during hemoglobin analysis, and that correctly characterize subjects carrying β-thalassemia.

Suspicion of mutation in delta globin should be raised when Hb A_2_ levels are below 2%. In the presence of another Hb A_2_-X peak, in order to evaluate the possible presence of masked β-thal defects, we need to sum the values of the components Hb A_2_ and Hb A_2_-X, to verify if Hb A_2_ values are in the normal range.

Low levels of Hb A_2_ are known to also be due to iron deficiency which represents the main cause of microcytosis and anemia in the population.

It has been observed that Hb A_2_ levels can be moderately reduced in patients with iron deficiency due to preferential binding of the heme with the β-chains, rather than with the δ-chains [25], and this reduction is even more evident with some variants of the δ-chains, as we have seen in Case 2. Therefore, subjects with low Hb A_2_ should always be examined to evaluate serum iron levels.

It is important to note that, although the delta variant is clinically not significant, the comprehensive analysis of hematology parameters and molecular testing will help in detecting the carrier status, and therefore will improve genetic counselling and possible prenatal diagnosis.

Characterization of novel variants of delta globin chains contributes to the expertise of laboratory professionals in the use of separative systems, such as HPLC and CE.

Although thalassemia and, in general, all hemoglobinopathies have today better therapeutic solutions than in the past, with less demanding transfusions and adequate iron chelation, with bone marrow transplantation or, very recently, with gene therapy in young subjects, prenatal diagnosis and preimplantation genetic diagnosis are still options available for a couple at risk of hemoglobinopathy. Therefore, a wide-ranging accurate molecular characterization is essential and a precision diagnostic approach is increasingly required, also to support personalized therapeutic pathways. However, all of this always starts from an accurate screening of carriers of thalassemia or clinically important hemoglobin variants, as happens in higher risk populations, such as the Italian one.

The use of new molecular methods with high sensitivity can allow for discovery in the future of many new hemoglobin variations, even asymptomatic, but that in compounds with thalassemic defects can determine complex phenotypes that must be appropriately considered within dedicated genetic counselling.

## Figures and Tables

**Table 1:** Hematological data and molecular results of the 8 new mutations of the δ-globin gene found in 12 subjects.

δ Variant (Name)	Sex-Age	RBC (×10^12^/L)	Hb (g/dL)	MCV (fL)	MCH (pg)	Hb A_2_ (%)	Hb A_2_X (%)	Iron Level	HBD HGVS Nomenclature	Other Globin Mutations	Case
δ130(-T) (Hb A_2_-Gaslini1)	F-35	4.66	12.0	81.0	27.0	1.6	n.d.	Normal	*HBD*:c.391delT		1
δ36(C2)Pro > Arg (Hb A_2_-Sanremo)	F-26	5.16	9.8	64.7	19.0	1.5	n.d.	Low	*HBD*:c.110C > G		2
	M-58	4.69	14.1	87.6	30.1	1.5	0.5	Normal	*HBD*:c.110C > G		3
δ3(NA3)Leu > Pro (Hb A_2_-Sile)	F-26	4.20	13.0	90.8	31.1	1.7(a)	n.d.	Normal	*HBD*:c.11T > C		4
δ74(E18)Gly > Asp (Hb A_2_-Asti)	M-10	6.87	10.8	56.8	15.7	2.7	n.d.	Normal	*HBD*:c.224G > A	*HBB*:c.118C > T (cd39 C > T)	5
	M-45	5.73	17.3	91.1	30.2	1.0	n.d.	n.d.	HBD:c.224G > A	*HBA2*:c.618C > A (Hb J Rovigo)	6
δ144(HC1)Lys > Glu (Hb A_2_-Angola)	F-33	3.93	11.8	82.2	28.2	2.0	n.d.	n.d.	*HBD*:c.433A > G	*HBB*:c.20A > T (HbS)	7
δ87(A4)Gln > Arg (Hb A_2_-Cremona)	F-40	4.43	12.7	86.8	28.7	1.5	1.0	Normal	*HBD*:c.263A > G		8
δ7(A4)Glu > Asp (Hb A_2_-Pordenone)	F-62	5.61	13.8	78.7	24.7	1.3	1.3	Normal	*HBD*:c.24G > C		9
	F-80	4.91	14.1	90.4	28.7	1.6	1.4	Normal	*HBD*:c.23A > C		10
δ7(A4)Glu > Ala (Hb A_2_-Udine)	M-54	5.86	14.0	65.6	23.8	2.8	2.2	Normal	*HBD*:c.23A > C	*HBB*:c.118C > T (cd39 C > T)	11
	F-26	5.28	13.3	78.8	25.1	1.8	1.2	n.d.	*HBD*:c.23A > C		12

n.d.: values not detected; (a): Hb A_2_-Normal + Hb A_2_-Sile.

## Data Availability

All of the eight new variants of the delta globin gene were submitted to publicly accessible repositories and openly available online: http://www.ithanet.eu and http://globin.cse.psu.edu/globin/hbvar/; https://globin.bx.psu.edu/hbvar/menu.html (HBVAR); https://www.lovd.nl/ (accessed on 31 October 2021).
